# Analysis of the Behavior of Mitochondria in the Ovaries of the Earthworm *Dendrobaena veneta* Rosa 1839

**DOI:** 10.1371/journal.pone.0117187

**Published:** 2015-02-11

**Authors:** Justyna Faron, Tytus Bernaś, Hanna Sas–Nowosielska, Jerzy Klag

**Affiliations:** 1 Department of Animal Histology and Embryology, University of Silesia, Katowice, Poland; 2 Lab. for Imaging Tissue Structure and Function, Nencki Institute of Experimental Biology, Warsaw, Poland; Hokkaido University, JAPAN

## Abstract

We examined six types of cells that form the ovary of the earthworm *Dendrobena veneta* ogonia, prooocytes, vitellogenic oocytes, trophocytes, fully grown postvitellogenic oocytes and somatic cells of the gonad. The quantitative stereological method revealed a much higher “volume density” of mitochondria in all of the types of germ-line cells except for the somatic cells. Fluorescent vital stain JC-1, however, showed a much higher oxidative activity of mitochondria in the somatic cells than in the germ-line cells. The distribution of active and inactive mitochondria within the studied cells was assessed using the computer program ImageJ. The analysis showed a higher luminosity of inactive mitochondria in all of the types of germ-line cells and a higher luminosity of active mitochondria in somatic cells. The OXPHOS activity was found in somatic cells mitochondria and in the peripheral mitochondria of the vitellogenic oocytes. The detection of reactive oxygen species (ROS) revealed a differentiated distribution of ROS in the different cell types. The amount of ROS substances was lower in somatic cells than in younger germ-line cells. The ROS level was also low in the cytoplasm of fully grown postwitellogenic oocytes. The distribution of the MnSOD enzyme that protects mitochondria against destructive role of ROS substances was high in the oogonia and in prooocytes and it was very high in vitellogenic and postvitellogenic oocytes. However, a much lower level of this protective enzyme was observed in the trophocytes and the lowest level was found in the cytoplasm of somatic cells. The lower mitochondrial activity and higher level of MnSOD activity in germ-line cells when compared to somatic cells testifies to the necessity of the organisms to protect the mitochondria of oocytes against the destructive role of the ROS that are produced during oxidative phosphorylation. The protection of the mitochondria in oocytes is essential for the transfer of healthy organelles to the next generation.

## Introduction

Numerous studies on somatic tissues that have been carried out since the second half of the 20^th^ c. have shown a direct connection of mitochondrial activity with their quantity (volume density, i.e. the percentage of the cell volume that is occupied by mitochondria) in the cytoplasm of a cell [1, 2, 3, 4, 5, 6, 7]. The studies have shown that higher number of mitochondria is connected with higher energy requirement of the cell [8, 9]. Muscle cells, nerve cells or liver cells have a higher relative volume density of mitochondria than bone or cartilage cells. Based on these assumptions some researchers have studied germ-line cells in an attempt to interpret the energy consumption of those cells by studying mitochondria distribution within a cell [10, 11] or by determining the relative volume of mitochondria in the cytoplasm [12, 13, 14, 15]. As a result, they have found a much higher volume density of mitochondria in germ-line cells than in somatic cells and it has been suggested that the energy requirements of germ-line cells might be higher than those of somatic cells. Such an interpretation of stereological results corresponds with the “disposable soma theory”, which claims that all of the syntheses in germ-line cells must be very accurate in order to avoid the accumulation of defective molecules and to keep the cells fit for further generations. Therefore, germ-line cells should possess more mitochondria than somatic cells because such a strategy requires high energy consumption [16]. Recently, studies using substances that directly show the level of mitochondrial activity have provided us with contradictory results. Some studies on germ-line cells have reported a high level of activity of mitochondria in *Xenopus* and zebrafish oocytes [17, 18] or in the germinal plasm in *Drosophila* embryos [10] while other studies that were recently carried out on human, mouse, bovine and, *Xenopus* oocytes and eggs using confocal microscopy and substances that indicate the level of mitochondrial activity have shown that the mitochondria in germ-line cells behave in a different way than the mitochondria in somatic cells and that during oogenesis or at least at certain stages of this process, a large number of these organelles remains inactive [19, 20, 21, 22, 23]. Moreover, the studies that have been carried out on the early embryos of different mammals have also confirmed that the majority of mitochondria remain inactive not only in germ-line cells but also in some blastomeres and embryoblast cells [19, 22, 23, 24, 25]. The data from invertebrates are few and scarce [10, 43].

There are several chemicals that can be used for the assessment of mitochondrial activity in living cells. Rhodamine-123, Mitotracker, DiOC_6_ and JC-1 have been used most frequently. The substances must be used very carefully in order to avoid the misinterpretation of results [26, 27, 69]. JC-1 (5,5′,6,6′-tetrachloro-1,1&rsquo;3,3′-tetraethylbenzimidazolcarbocyanine iodide) was the most suitable for our study because it can show both the active and inactive mitochondria in the same cell. After entering into the mitochondria, it changes color from green (monomeric form—inactive mitochondria) to red as the mitochondrial membrane becomes polarized and the aggregates of the JC-1 stain form (J-aggregate—active mitochondria). Therefore, we decided to use this substance to study the activity of the mitochondria in the gonads of the annelid *Dendrobaena veneta*. In addition, we used stereological methods to assess the volume density of these organelles in the gonads of the annelid in order to confirm the results for Collembola embryos that were presented in our previous papers [13, 14]. This annelid species is suitable for studies of the activity of mitochondria in gonads because its’ ovaries are meroistic and contain five types of germ-line cells (oogonia, prooocytes, trophocytes, oocytes, and postvitellogenic oocytes) as well as somatic cells that fill up all of the spaces among the germ cells [28]. The mitochondrial activity of a species with this type of gonads has not been studied until now. Moreover, we decided to compare the activity of mitochondria in germ and somatic cells by detecting the activity of cytochrome oxidase, as well as to look for the distribution of reactive oxygen species (ROS) in the gonad and for the presence of mitochondrial superoxide dismutase (MnSOD) in order to assess the threat from ROS and the defense mechanisms against these substances in the different cell types.

## Material and Methods

### Electron microscopy

The ovaries of sexually mature specimens of the earthworm *Dendrobena veneta* Rosa, 1839 with a well-developed clitellum were examined. The ovaries were dissected from worms using a stereoscope microscope and were then prepared for the following studies.

For electron microscopy the ovaries were fixed using 2% OsO_4_ (at 4°C overnight). After being rinsed in a 0.1M phosphate buffer (pH 7.4), they were dehydrated in increasing concentrations of alcohol and acetone. The material was then embedded in epoxide resin Epon 812. Semithin sections were cut with a TESLA BS 490A ultramicrotome and stained with 1% methylene blue in 0.5% borax. The sections were analyzed and photographs were taken under an OLYMPUS BX60 light microscope with a DP12 digital camera and the AnalySIS 3.2 computer program.

For the electron microscopy epon blocks were cut using a LEICA UTC25 ultramicrotome and diamond knives. The ultrathin sections were contrasted with uranyl acetate and lead citrate and analyzed under a HITACHI H500 electron microscope.

### Stereology

Stereology is used to study three-dimensional objects through the interpretation of two-dimensional images. This is useful not only because it allows the structure of entire cells and tissues to be studied based on thin sections or photomicrographs of sections, but also because it allows these structures to be studied quantitatively [29]. Using this method it is possible to assess the relative volume (in the percentage) of a given type of organelles within a cell (the relative density of organelles in a cell) and then to compare this parameter in different cell types. The proportions of the values that are counted on the surface directly relate to the proportions of volumes [30, 31, 32].

Fifty photographs (magnification 10,000x) were taken for each of the five cell types (oogonia, prooocytes, trophocytes, vitellogenic oocytes and somatic cells) that are present in the worm’s ovary. The pictures were then analyzed using Delesse’s (1848) method. A grid of 100 squares was imposed on each picture using the ImageJ computer program. In order to assess the relative volume of the organelles, the points of the intersections of the grid lines that lay on the chosen structures were counted and compared to the remaining points. Mitochondria, Golgi bodies, nucleuses and the ground cytoplasm were taken into account. The results were analyzed using the nonparametric Anova test (Kruskal-Wallis) p<0.05 using the Statistica 8 computer program.

### Detection of mitochondrial activity

Mitochondrial activity was studied using the fluorescent dye JC-1 (5,5′,6,6′-tetrachloro-1,1′,3,3′-tetraethylbenzimidazolcarbocyanine iodide), which acts as a marker of mitochondrial activity and allows changes in the electrochemical potential of inner mitochondrial membrane to be detected. Dissected ovaries (1mm long, less than 0.5 mm thick) were rinsed in PBS and immersed in a solution with the JC-1 (1mg/ml DMSO) (Sigma) in PBS (10μl JC-1 in 2 ml PBS for 60 minutes). Both monomeric (excitation at 488nm, emission 500–550nm) as well as aggregation (excitation 488 nm, emission at 575–620nm) were registered using an Olympus FV1000 confocal system combined with an Olympus IX81 inverted confocal microscope with a 60x PlanApo water objective. The images were analyzed using the ImageJ program. Image segmentation was performed using the Multi-Otsu threshold plug-in for the ImageJ program. In order to perform the image segmentation the histogram of each image was first extended to saturate the gray scale. Then, the images were filtered using a median filter (r = 0.5). Afterwards 12-bit images were converted to an 8-bit gray scale. An algorithm then attributed pixels to the appropriate class of luminosity (255 steps of luminosity) which was arbitrarily divided into: class 1: 1 to 50; class 2: 51 to 100; class 3: 101 to 150; class 4: (the brightest): 151 to 255.

### Detection of cytochrome oxidase activity (according to [80])

Applying DAB (3,3′-diaminobenzidine) as the electron donor allows for observation of an insoluble reaction product in the place where cytochrome oxidase is active, using an electron microscope. Isolated ovaries were fixed in a mixture of 2.5% of formaldehyde with 1.5% of glutaraldehyde dissolved in a 0.1M phosphate buffer with the addition of 4% saccharose over 60 minutes. After rinsing overnight in a 0.1M phosphate buffer with saccharose, the tissue was incubated (3 h. at 37°C in the dark) in this mixture: phosphate buffer + saccharose + DAB (Polysciences) and cytochrome-c (Sigma). The control reaction was done by incubating the ovaries with an incubation mixture with 0.05M KCN—a cytochrome oxidase inhibitor added. The tissue was washed in a phosphate buffer with saccharose and post-fixed in 2% Osmium tetroxide and embedded in Epon 812 after dehydration.

### Detection of Reactive Oxygen Species (according to [81])

Dissected ovaries were rinsed in PBS with 0.0025% triton X100 and pure PBS and stained with 30μm DHE (dihydroethidine—Invitrogen) in PBS. The stain was prepared from a 30mM stock solution of DHE in anhydrous DMSO. After being rinsed in PBS, the preparation was embedded in a VECTASHIELD medium. The analysis was performed using an Olympus IX81 confocal microscope.

### Detection of mitochondrial superoxide dismutase (MnSOD)

Immunofluorescence

Dissected ovaries that had been embedded in Tissue-Tek O.C.T. Compound (Electron Microscopy Sciences) were cut on a Tissue-Tek II cryostat. The cryostat sections were cut (5μm of thickness) and mounted on slides. After being rinsed in TBS and a solution of 0.1% triton X100 in TBS the slides were incubated (2 hours at room temperature) in a 2% albumin solution in TBS with 0.1% Triton. After desiccation, the slides were incubated in an active MnSOD antibody (Rabbit Anti-MnSOD Polyclonal Antibody—Stressgen) overnight at room temperature. On the following day the slides were washed in TBS and TBS with 0.1% triton and incubated in goat Anti-Rabbit IgG Biotin Conjugate (Sigma) (1.5 h at room temperature). After washing in TBS the material was stained with streptavidin-fluorescein conjugated with FITC (GE Healthcare) for 30 minutes and finally rinsed in TBS. Sections were examined using an OLYMPUS BX60 fluorescence microscope.

Immunogold method

Dissected ovaries were fixed in a mixture of 2.5% glutaraldehyde and 4% formaldehyde (1:1) in TBS and eventually rinsed in TBS. After dehydration in an alcohol gradient until absolute alcohol the ovaries were embedded in LR White. The material was cut into ultrathin sections and placed on nickel mesh and incubated with Rabbit AntiMn SOD Polyclonal Antibody (Stressgen) followed by Colloidal Gold Conjugated Goat Anti-rabbit IgG (SPI Supplies and Structure Probe). The sections were then contrasted with uranyl acetate and lead citrate and analyzed using a HITACHI H500 electron microscope.

## Results

### Ovary structure and oogenesis in *Dendrobaena veneta*

**Fig 1:**
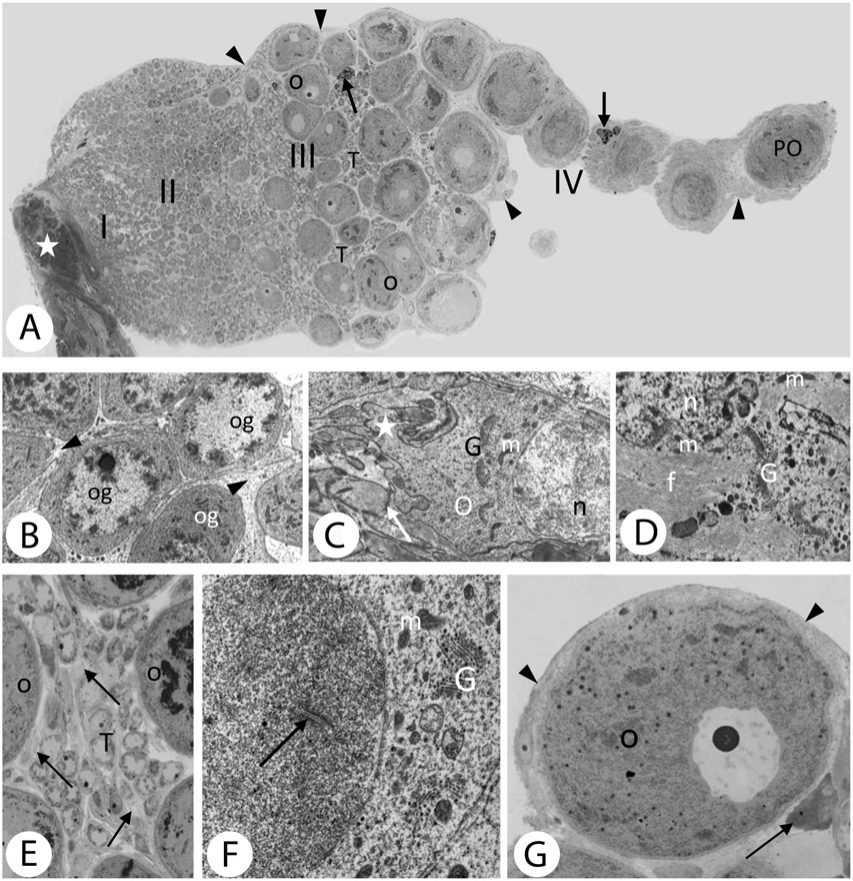
Morphology of *Dendrobaena veneta* ovary showing the ultrastructure of all cell types building the earthworm’s gonad. *A*. Longitudinal section of the ovary with the attachment site to the intersegmental wall (asterisk). Roman numbers indicate the 4 zones of the gonad: zone I – close to the attachment site contains oogonia; zone II – where differentiation of the prooocytes occurs; zone III – previtellogenesis and vitellogenesis with the growing oocytes (O) and the trophocytes (T); zone IV – where postvitellogenic oocytes (PO) detach from the ovary to swim freely in the coelomic fluid. Degenerating trophocytes (arrows) are embedded in the somatic cells (arrowheads) surrounding the oocytes. All germ-line cells are immersed within the framework made by the somatic cells. Light microscopy, semithin section, magn. x240. *B*. Oogonia (og) with almost spherical nuclei with characteristic clumps of chromatin are tightly enveloped by somatic cells (arrowheads). TEM, magn. x6700. *C*. In the cytoplasm of the young oocyte (O) with nucleus (n), numerous Golgi bodies (G) and mitochondria (m) are visible. The oocyte is connected to other members of the cluster (trophocytes) by a cytoplasmic cord (asterisk). The oocyte is surrounded by somatic cells that are firmly connected by extensive desmosomes (arrow). TEM, magn. x6600. *D*. The somatic cell of the gonad. Beside nucleus (n), Golgi bodies (G) and a few mitochondria (m), there is a huge number of intermediate filaments (f) in its’ cytoplasm. TEM, magn. x18800. *E*. Among the growing oocytes (O) of zone III of the ovary, there are numerous trophocytes (T) and some somatic cells (arrows). Light microscope, semithin section, magn. x1300. *F*. Pro-oocyte with synaptonemal complexes in the nucleus (arrow), mitochondria (m) and Golgi bodies (G) in the cytoplasm can be seen. TEM, magn. x20000. *G*. Postvitellogenic oocyte (O) of zone IV of the ovary surrounded by some layers of the somatic cells (arrowheads). A group of degenerating nurse cells is visible close to the oocyte (arrow). Light microscope, semithin section, magn. x1200.

Oogenesis in the earthworm *Dendrobaena veneta* takes place in one pair of ovaries that are situated in the 13^th^ body segment. They are small, slightly flattened structures that are attached to the intersegmental wall. The main body of the gonad is composed of somatic cells (Fig. 1D, G) whose projections embrace the germ-line cells tightly (Fig 1B, C, G).

Four zones can be discerned in the ovary (Fig. 1A). Zone I contains proliferating oogonia (Fig. 1B). After leaving zone I, the germ-line cells enter zone II where they enter meiosis and become prooocytes (Fig. 1F). The prooocytes differentiate into oocytes or into trophocytes in zone II (Fig. 1E). Both types of germ-line cells that arise from a single oogonium remain connected by cytoplasmic cords. Previtellogenesis occurs in zone III and vitellogenesis takes place close to the zone IV. The oocytes then become much bigger than the trophocytes (Fig. 1E). At the free end of the ovary there are huge postvitellogenic oocytes wrapped with several layers of somatic cells in zone IV, (Fig. 1A, G), and after losing the cytoplasmic connections with the oocytes the trophocytes eventually degenerate (Fig. 1A, G). When oogenesis is accomplished, the fully grown oocytes are released into the coelomic fluid.

The somatic cells of the gonad are present in all four zones of the ovary. They send numerous projections that wrap around individual germ-line cells into the interior of the gonad (Fig. 1B, D, G). For a detailed description of the gonad in *Dendrobaena veneta* see [28].

### Stereological analysis

**Fig 2:**
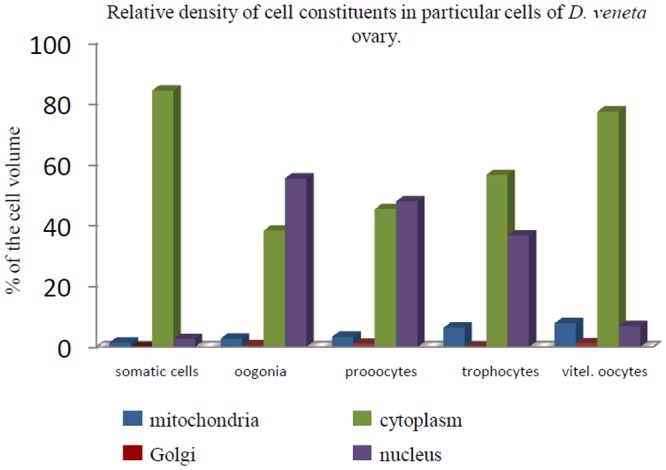
The graph shows the results of stereological studies of the constituents of the cells in *D*. *veneta* gonads: mitochondria, cytoplasm, Golgi body and nucleus in particular cells (somatic cells, oogonia, prooocytes, trophocytes and vitellogenic oocytes). The X coordinate shows the relative volume of a given cell constituent.

Among the cells that comprise the gonad of the earthworm, the somatic cells had the lowest relative volume of mitochondria, i.e. 1.44%. The relative volume of these organelles in germ-line cells changed with the progress of oogenesis. The relative volume of mitochondria in oogonia was 2.77%, in prooocytes 3.31%, in trophocytes 6.31% and in vitellogenic oocytes that value was 7.78% (Fig. 2, Table 1).

**Table 1:** Numerical data of stereological results.

	% mito	% aG	% nu	% cyto
SOM	1,44	0,04	2,65	84,43
OG	2,77	0,47	55,50	38,26
PRO	3,31	1,11	47,93	45,37
TRO	6,31	0,10	36,67	56,62
VO	7,78	1,30	6,91	77,53

Numerical data of the stereological results showing parts of the cell volume that is occupied by: mito – mitochondria, aG – Golgi bodies, nu – nucleus and cyto – cytoplasm in particular cells of the *D*. *veneta* gonad. SOM – somatic cells, OOG – oogonia, PRO – prooocytes, TRO – trophocytes, VO – vitellogenic oocytes.

Stereological analysis of the relative volumes of Golgi bodies in somatic cells and four types of germ-line cells showed very low values of this parameter. The highest level was found in the vitellogenic oocytes (1.3%) and the prooocytes (1.11%). The values were very low in the remaining cells (somatic cells—0.04%, oogonia—0.47%, trophocytes—0.1%) (Fig. 2, Table 1).

The relative volume of the cytoplasm that was free from organelles in somatic cells was the highest among all gonad’s cells and attained 84.43% of the cell volume. The relative volume of the cytoplasm in the germ-cells of the gonad gradually increased with advancing oogenesis from 38.26% in oogonia, 45.37% in prooocytes, 56.62% in trophocytes to 77.53% in the vitellogenic oocytes (Fig. 2, Table 1).

The relative volume of the nucleus behaved in a different manner. It was the highest in oogonia (55.5%), lower in the prooocytes (47.93%) and the trophocytes (36.67%) and the lowest in the vitellogenic oocytes, where it dropped to 6.91% of the cell’s volume. The relative volume of the nucleus in the somatic cells was far below the lowest value for germ-line cells and was as low as 2.65% (Fig. 2, Table 1).

### Activity of mitochondria

The experiment using the fluorescent dye JC-1, which is sensitive to the mitochondrial membrane potential and that shifts from green to red with increasing membrane potential allows for a dual color (green/red) semi-quantitative assessment of the mitochondrial polarization states. In this way it is possible to show the differences in mitochondrial activity in a tissue using the same preparation. The differences of the membrane potential across the inner mitochondrial membrane are essential for the activity of these organelles. A high membrane potential allows for the formation of dye aggregates that give a red fluorescence of the active mitochondria, while a low membrane potential gives a green fluorescence of the monomeric dye form.

**Fig 3:**
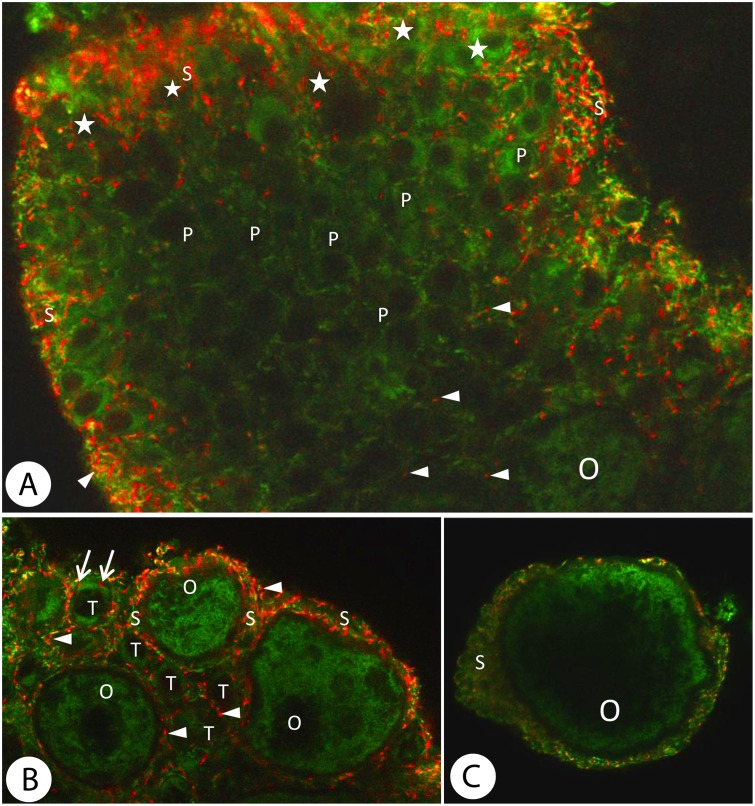
Merged images of *Dendrobaena veneta* ovary treated with JC-1 cationic stain showing all cell types buiding the gonad. The green fluorescence shows inactive and the red one active mitochondria in the cells. *A*. Zones I and II (and part of the zone III with previtellogenic oocytes (O) of a JC-1 stained gonad. A green fluorescence can be seen in the cytoplasm of both the oogonia (asterisks) and the prooocytes (P). A red one prevails in the somatic cells (arrowheads). Confocal microscope, JC-1 staining, magn. x600. *B*. Zone III. Previtellogenic oocytes (O) and trophocytes (T) immersed in a network of follicular cells (S). The germ-line cells express green fluorescence which testifies to the decreased activity of their mitochondria. In some trophocytes individual red marks are visible (arrows). The somatic cells (arrowheads) express the strong red fluorescence of highly active mitochondria. Confocal microscope, JC-1 staining, magn. x800. *C*. The oldest vitellogenic oocyte from zone IV surrounded by a few layers of somatic cells (S). The green fluorescence of inactive mitochondria dominates in the oocyte (O) while the red fluorescence of active mitochondria is mainly visible in the somatic cells. Confocal microscope, JC-1 staining, magn. x720.

**Fig 4:**
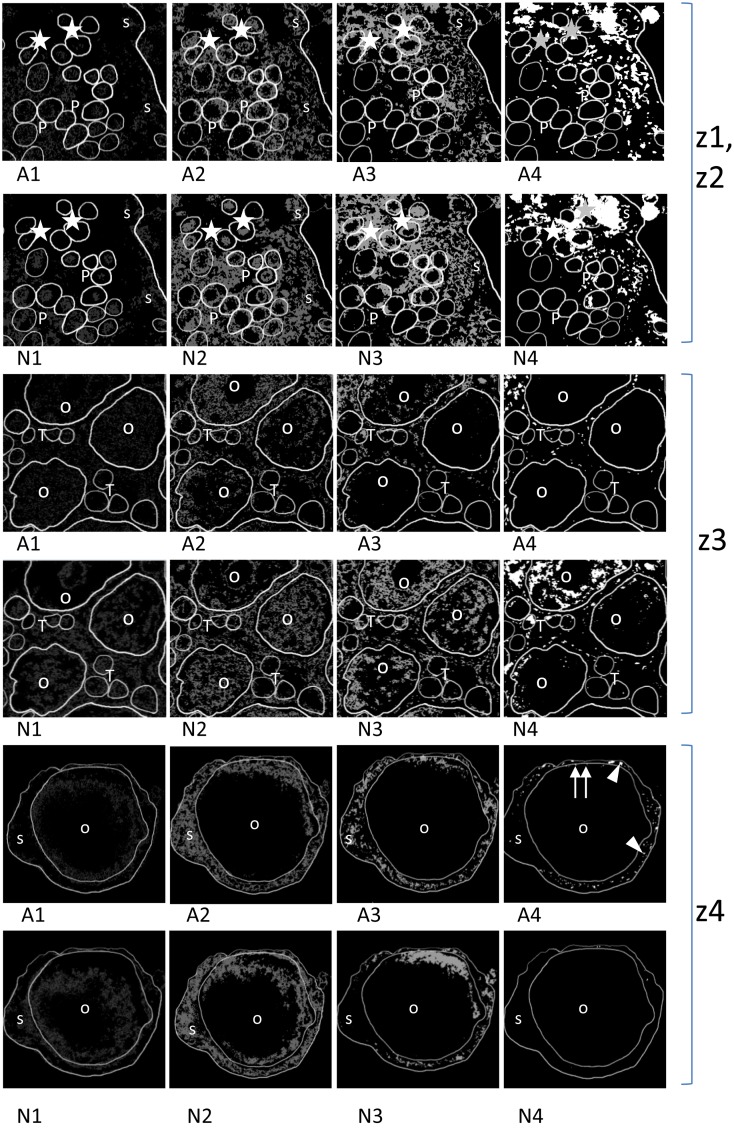
Three sets of image segmentation of the JC-1 stained ovary fragments performed using the Multi Otsu Threshold plug-in for the ImageJ computer program. Zone I and zone II (z1, z2). Two rows of pictures showing the image segmentation of the JC-1 stained ovary fragments. The upper row shows the four classes of red fluorescence intensity (A1 to A4); the lower row shows the four classes of green fluorescence intensity (N1–N4). In oogonia (asterisks) A1 to A3 activity is seen, A4 is present in some of the gonial cells. In prooocytes (P) mainly A1 and A2 activity is visible. A3 is seen only in some cells and A4 is almost absent. In oogonia (asterisks) inactive mitochondria are seen in all four classes of fluorescence intensity (N1 to N4) although the latter is distributed irregularly. In prooocytes (P) inactive mitochondria are distributed in entire cytoplasm (N1 to N3) but inactive mitochondria of N4 class are distributed so irregularly that they are seen only in some cells (arrows) on the presented section. In somatic cells both A1 and N1 fluorescence are low but A2 to A4 and N2 to N4 are expressed with a similar intensity. Confocal microscope, JC-1 staining, magn. x500. Zone III (z3). Two rows of pictures showing the image segmentation of the JC-1 stained ovary fragments. The upper row shows the four classes of red fluorescence intensity (A1 to A4); the lower row shows the four classes of green fluorescence intensity (N1–N4). In the oocytes (O) fluorescence intensity of A1 and A2 classes are seen. Activity of A3 class is weaker and A4 is absent. In the lower row the fluorescence intensity of inactive mitochondria of N1 to N3 classes is strong and N4 class is also noticed. In the trophocytes (T) the fluorescence intensity of A1 to A3 classes is visible and in the class A4 single signals are noticed. In the green fluorescence the fluorescence of all N1 to N4 classes occur. In somatic cells both A1 and N1 fluorescence are clearly visible and A2 to A4 and N2 to N4 are expressed with similar intensity. Confocal microscope, JC-1 staining, magn. x600. Zone IV (z4). Two rows of pictures showing the image segmentation of the JC-1 stained ovary fragments. The upper row shows the four classes of red fluorescence intensity (A1 to A4), the lower row shows the four classes of green fluorescence intensity (N1–N4). In the vitellogenic oocyte (O) fluorescence intensity of A1is visible almost regularly in ooplasm and A2 occurs in the subplasmalemmal position. Activity of A3 class is restricted to one of the cell poles and A4 gives individual signals (arrows). In the lower row the intensity of inactive mitochondria of class N1 is restricted to the internal cytoplasm, the class N2 is situated in the outer part of the ooplasm and N3 is shifted to one of the poles but N4 is absent. In somatic cells (s) both A1 and N1 fluorescence are low, A2 and N2 are very similar but A3 and N3 are almost identical. While A4 is apparent (arrowheads) N4 is almost absent. Confocal microscope, JC-1 staining, magn. x400.

Merged images showing both high-polarized and low-polarized mitochondria in the same picture indicate the relative proportion of active to inactive mitochondria in a given cell. In our images one can see mostly red fluorescence in the somatic cells and mostly green fluorescence in the germ-line cells (Fig. 3A, B, C). Therefore, these images show a much higher mitochondrial activity in the somatic cells and a much lower activity of these organelles in the germ-line cells. To allow for a quantitative analysis of the intensity of the reaction using the Multi Otsu Threshold plug-in for the ImageJ program, the fluorescence spectrum in both spectrum channels was divided into four intensity classes (see Material and Methods). The distribution of the mitochondria with different activity in gonad cell types that were studied was classified in this way (Fig. 4).

The cells that form the gonad of *D*. *veneta* had different activity levels depending on their types. All cell types contained both active (expressing red light) and inactive (expressing green light) mitochondria. Somatic cells appeared to have a much higher intensity of red fluorescence (A1—A4) and a much higher proportion of active mitochondria in relation to inactive ones (N1—N4) than any type of germ-line cells. The activity of mitochondria in somatic cells that was measured as the intensity of red fluorescence appeared in classes A2, A3 and A4 (Fig. 4 z1, z2, Table 2). The activity of class A1 was not strong in somatic cells. Similarly, inactive mitochondria (N) expressed their light intensity in classes N2 to N4 (Fig. 4 z1, z2, Table 2).

**Table 2:** Quantity of active mitochondria (in classes A1–A4) and inactive (N1–N4).

	A1	A2	A3	A4	N1	N2	N3	N4
SOM	++	++++	++++	+++	+	++++	++++	+++
OOG	+	++++	++++	+	+	++++	++++	+++
PRO	++++	++++	+		++++	++++	+++	+
TRO	++++	++++	++	+	++++	++++	+++	++
VO	++++	++++	+		+++	++++	+++	++
PO	++	++	+		+++	+++	++	

Mitochondrial activity (JC-1 staining) in classes A1–A4 and inactive in classes N1–N4 in the cells that form the ovary in *D*. *veneta*. The number of crosses indicates the approximate density.

The active mitochondria in germ-line cells appeared only in fluorescence intensity classes A1 to A3 and rarely or not at all in class A4 although inactive mitochondria were seen in all four classes of fluorescence intensity (N1–N4). In the oogonia that occupied zone I of the ovary (Fig. 4 z1, z2) both active and inactive mitochondria expressed the fluorescence intensity of classes 1, 2 and 3 but inactive mitochondria also expressed class 4 of this parameter, which testifies to the low activity of the majority of these organelles. Active mitochondria of class A4 with red fluorescence appeared in some oogonia. All of the mitochondria in oogonia were distributed regularly in the cytoplasm (Fig. 4 z1, z2, Table 2).

Prooocytes (Fig. 4 z1, z2) that contained active mitochondria with a red fluorescence intensity were restricted to classes A1, A2 and A3 while mitochondria with a low electrochemical potential appeared in classes N1, N2, N3 and N4 of green fluorescence. All of the mitochondria in these cells were distributed regularly around the nucleuses. The trophocytes expressed a strong green fluorescence of inactive mitochondria in classes N1, N2, N3 and N4. The majority of active mitochondria in the trophocytes expressed their red fluorescence in classes A1 and A2, much less in A3 and exceptionally one could see isolated points in class A4. The majority of the organelles in these cells were gathered at one cell pole and the rest were rarely dispersed in the cytoplasm (Fig. 4 z3, Table 2).

The red fluorescence of active mitochondria in vitellogenic oocytes was much lower than the green fluorescence. There were signals of classes A1 and A2, isolated signal points of A3 class but fluorescence of class A4 was practically absent. Instead, the signal of inactive mitochondria of classes N1 to N3 were strong and there were numerous signals coming from many areas of the ooplasm in class N4 (Fig. 4 z3, Table 2).

The activity level of mitochondria in postvitellogenic oocytes just before ovulation was low (Fig. 4 z4). The active mitochondria in classes A1 and A2 were distributed throughout the cytoplasm but rather at the periphery of the cell. The organelles in class A3 of light intensity were not numerous and were shifted towards one cell pole creating a crescent-like accumulation. The mitochondria were practically absent in class A4. Inactive mitochondria that fell into class N1 filled up almost all of the cytoplasm volume except the cell pole that was occupied by the A2 mitochondria. Numerous mitochondria of class N2 occurred in the outer periphery of the ooplasm. Mitochondria N3 occupied the region close to the A3 crescent but they were much more numerous. The mitochondria N4 were absent. This pattern of mitochondria distribution was found in all of the vitellogenic oocytes that were scanned (Fig. 4 z4, Table 2).

### Detection of Cytochrome Oxidase (EC 1.9.3.1)

**Fig 5:**
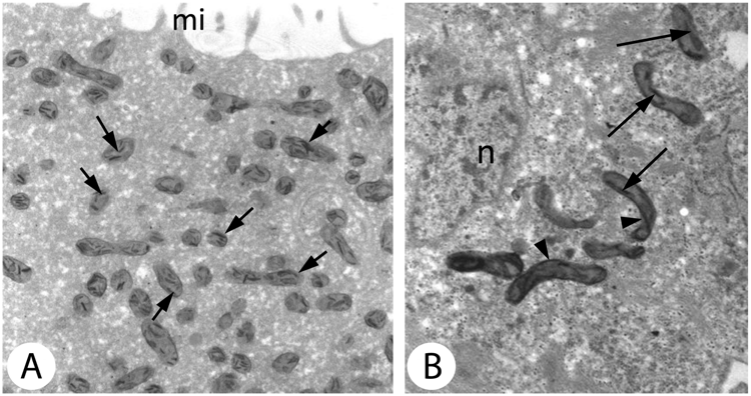
Detection of cytochrome oxidase activity within the mitochondrial cristae. *A*. The peripheral regions of a vitellogenic oocyte (arrows). mi—microvilli on the oocyte surface. TEM, DAB reaction, magn. x20000. *B*. Cytochrome oxidase activity within the mitochondrial cristae (arrows) and in the intermembrane space (arrowheads) of a somatic cell of the gonad. n—nucleus. TEM, DAB reaction. magn. x18700.

DAB was applied as an electron donor for the cytochrome c and made it possible to visualize insoluble reaction products under an electron microscope, on those mitochondria membranes where the OXPHOS activity took place. This method was applied in somatic cells and postvitellogenic oocytes. In the mitochondria of somatic cells, the reaction products were found at the level of mitochondria cristae and in the inter-membrane spaces in the outer fragments of the organelles (Fig. 5B). In the postvitellogenic oocytes, the reaction product was mainly found in the inter-membrane spaces of the mitochondria cristae (Fig. 5A). The reaction was observed in groups of mitochondria that were situated in the peripheral fragments of the ooplasm. No OXPHOS activity was found in the organelles that were situated in the interior of the ooplasm.

### Detection of Reactive Oxygen Species (ROS)

Dihydroethidine (DHE) is a fluorescent dye that, after being oxidized by reactive oxygen species, intercalates into DNA of living cells and emits red fluorescence.

**Fig 6:**
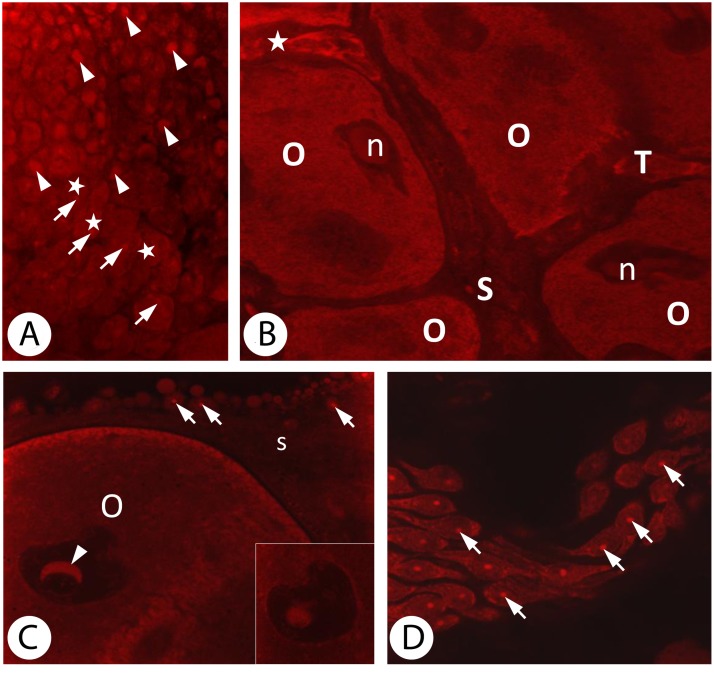
Detection of ROS in all the ovary constituents. *A*. Zone II of the ovary. In some prooocytes the signal is emitted by the entire nucleus (arrowheads). In the other the signal is limited to a smaller nucleus region (arrows) and is also visible in the cytoplasm (asterisks). Confocal microscope, DHE staining, magn. x750. *B*. Zone III of the ovary. ROS detection. Nuclei (n) of the growing oocytes (o) emit the weakest signal among the ovary cells with a strong signal from the cytoplasm. Between oocytes there are somatic cells (S) with the smallest amount of reactive oxygen species. Among trophocytes (T) there are cells with a low amount of ROS but the degenerating trophocytes express a much stronger signal (asterisk). Confocal microscope, DHE staining, magn. x 760. *C*. ROS in a vitellogenic oocyte (O) and surrounding somatic cells (s). Characteristic crescent-shaped fragment of the nucleus (arrowhead). Another section plane shows the circular profile of the structure (inset). The signal from the cytoplasm is weaker than in younger oocytes. Somatic cells surrounding the oocyte emit a strong signal from the nuclei (arrows). Confocal microscope, DHE staining, magn. x1500. *D*. Somatic cells of the gonad emit a strong signal from the nuclei (arrows) and a much weaker one from the cytoplasm. Confocal microscope, DHE staining, magn. x2000.

The use of this reagent for *D*. *veneta* gonads revealed a diverse distribution of ROS within the prooocytes. A strong signal coming from the nucleus was seen in some of the cells while in others the signal from the nucleus was weaker and the signal from the cytoplasm stronger (Fig. 6A).

The level of ROS in the majority of the trophocytes was not high but there was apparently a higher signal emitted by an oxidized form of dihydroethidine in some of them (most probably those that were degenerating) (Fig. 6B).

The nucleuses of the vitellogenic oocytes emitted the weakest fluorescence signal of all of the cells that form the gonad. However, the amount of light emitted from the ooplasm indicated a higher amount of ROS reacting with the dye molecules that were bound to DNA of the mitochondria, which were numerous in those cells (Fig. 6B). A structure in the shape of a sphere or a crescent (Fig. 6C) (depending on the section plane—Fig. 6C—inset) was seen in the nucleuses of the largest postvitellogenic oocytes. The signal from the oocyte cytoplasm was much weaker in comparison to the younger oocytes. Long processes of the somatic cells localized between vitellogenic oocytes contained the smallest amount of ROS (Fig. 6B). The somatic cells lying around the postvitellogenic oocytes emitted a strong signal from their nucleuses and a much weaker signal from their cytoplasm (Fig. 6D).

### Detection of SuperOxide Dismutase (E.C. 1.15.1.1)

The immunofluorescent method for detecting the SuperOxide Dismutase (MnSOD) at the level of light microscope allowed to observe the expression of MnSOD in the mitochondria of the cells that form the *D*. *veneta* ovary.

**Fig 7:**
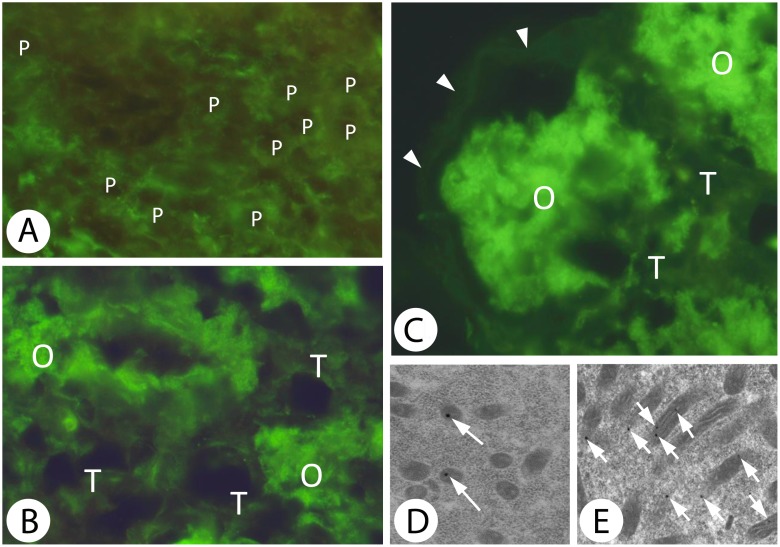
Detection of MnSOD enzyme using immunofluorescence method (the enzyme is detected thanks to green fluorescence) and immunogold labelling (black particles on the mitochondria profiles show the presence of the enzyme). *A*. MnSOD in Zone II of the *D*. *veneta* ovary. P—prooocytes express a low level of the enzyme that protects mitochondria against reactive oxygen species. Fluorescent microscope, staining using the antibody against MnSOD enzyme. Fluorescence microscope, immunofluorescent staining with anti—MnSOD antibody, magn. x750. *B*. MnSOD in Zone III of the *D*. *veneta* ovary. Mitochondria of the oocytes (O) give a much stronger fluorescence than those of the trophocytes (T). Fluorescent microscope, staining using the antibody against MnSOD enzyme. Fluorescence microscope, immunofluorescent staining with anti—MnSOD antibody, magn. x600. *C*. MnSOD in Zone III of the *D*. *veneta* ovary. Mitochondria of the oocytes (O) contain a large amount of the enzyme that protects the cells against ROS molecules. They express a much stronger fluorescence than the fluorescence of the somatic cells (arrowheads) and the trophocytes (T), which contain a much lower level of this enzyme. Fluorescent microscope, staining using the antibody against MnSOD enzyme. Fluorescence microscope, immunofluorescent staining with anti—MnSOD antibody, magn. x600. *D*. Numerous gold particles show MnSOD situated within the mitochondria of oocytes using immunogold labelling (arrows). TEM, immunogold labelling, magn. x30000. *E*. Immunogold labelling. Gold particles (arrows) showing MnSOD situated within the mitochondria of trophocytes are less numerous than those in the oocyte. TEM, immunogold labelling, magn. x17800.

The reaction revealed a moderate level of this enzyme in the prooocytes (Fig. 7A). The mitochondria of the oocytes expressed a higher amount of MnSOD than the organelles in the prooocytes and trophocytes (Fig. 7B). The lowest level of this enzyme was found in the somatic cells enveloping the vitellogenic oocytes (Fig. 7C). The expression of MnSOD in the trophocytes (Fig. 7D) and the oocytes (Fig. 7E) was confirmed at the electron microscope level using Goat Anti-rabbit IgG antibody coupled with colloidal gold particles. Higher density of MnSOD- gold labelled particles were present in the cytoplasm of vitellogenic oocytes rather than trophocytes.

## Discussion

In all of the papers that describe the results of stereological research of somatic tissues (see Introduction), one can find a good correlation between a cell’s activity and the volume density of its mitochondria [5, 8]. In a few cases stereological methods have been used for studying the volume density of mitochondria in germ-line cells. These studies revealed the presence of a higher volume density of mitochondria in germ-line cells than in somatic cells [12, 13, 14, 15]. According to the hypothesis that the volume density of mitochondria corresponds with the cell activity, the authors claimed that germ-line cells should be more active metabolically than somatic cells [12, 13, 14]. Some experimental results have indeed confirmed the higher activity of germ-line cells in mouse [33] or egg regions that contain germ-line cell determinants in fruit fly in comparison with somatic cells or ooplasm regions, which are destined to form the somatic parts of a future embryo [10, Table 3]. More recently, many papers have reported that in Xenopus, cattle and human at least a certain fraction of the mitochondria in germ-line cells remains inactive [17, 19, 21, 25, 34].

**Table 3:** Mitochondrial activity in the germ-line cells, somatic cells of the gonads and the embryos of different animal species studied to date.

mitochondria in:	Somatic cells of the gonad	PGCs	oogonia	trophocytes	previtellog. oocytes	vitellog. oocytes	postwitellog. oocytes	embryos
*Dendrobena*. *veneta—*meroistic ovary	mostly high activity	n.d.	mostly low activity; in dividing cells high activity	mostly low activity	mostly low activity	mostly low activity	mostly low activity; asymmetric distribution within the ooplasm	n.d.
mammals—follicular ovary,	n.d.	n.d.	n.d.	absent	n.d.	vitellogenesis absent	mostly low activity or anaerobic ATP production; asymmetric distribution of mitoch. within the ooplasm. [36, 34, 38, 55]	trophectoderm higher activity, embryoblast low activity [23, 36, 38]
*Drosophila melanogaster—*meroistic ovary	n.d.	active [10]	n.d.	n.d.	one mitoch. population enters the germ plasm, the other fills the ooplasm [35]	n.d.	mitochondria in the oosome region more active than in the ooplasm [10]	Mitoch. in the somatic cells less active than in the pole cells [10]
*X*. *laevis—*follicular ovary	n.d.	n.d.	n.d.	absent	mostly low activity [21]	“somatic” mitochondria active, “germ-line” mitochondria inactive [21, 48, 49]	“somatic” mitochondria active, “germ-line” mitochondria inactive [21, 49]	n.d.
*Danio rerio*—follicular ovary	n.d.	n.d.	n.d.	absent	fluorescence emitted by active mitoch. equals that of inactive ones [18]	fluorescence emitted by active mitoch. equals that of inactive ones [18]	n.d.	n.d.

Therefore, we decided to study the earthworm’s ovary using stereology as well as JC-1 staining to see if the stereology that was applied to the germ-line cells of *D*. *veneta* is acceptable to draw conclusions about a cell’s metabolism. JC-1 is a dye that allows to observe simultaneously active (red fluorescence) and inactive (green fluorescence) mitochondria in living cells [26, 27]. As the most of publications concern vertebrates this study can bring on some new data on invertebrates. Moreover, the meroistic ovary of *D*. *veneta* contains all stages of oogenesis from oogonia to the postvitellogenic oocytes and the trophocytes wraped by the somatic cells. The present paper shows for the first time the mitochondrial activity in all cell types building the merositic ovary in confrontation with the stereology.

### Stereology

The results of the present paper show a much higher volume density of mitochondria in all of the types of germ-line cells (oogonia, prooocytes, oocytes, trophocytes and postvitellogenic oocytes) and much lower within the somatic cells of the gonads. This parameter was the lowest in oogonia (2.77%) but was still much higher than in the somatic cells (1.44%). Moreover, the relative volume of these organelles increased during the differentiation of germ-line cells (3.31% in oocytes, 6.31% in trophocytes and 7.78% in postvitellogenic oocytes). Using the assumption that the volume density of mitochondria indicates the activity of these organelles, one could say that the energy consumption of germ-line cells grows along with the progress of gametogenesis. However, application of the fluorescent dye JC-1 in the ovaries of *D*. *veneta* revealed a completely different situation.

### Examination of mitochondrial activity using JC-1

The highest activity of mitochondria in *D*. *veneta* ovaries was found in the somatic cells of the gonad whereas the activity of mitochondria in all of the types of germ-line cells was suppressed considerably. This means that in this species there is a great discrepancy between the stereological results and the mitochondrial activity that was revealed using the fluorescent dye JC-1. Our results appear to be in agreement with papers that report the reduced activity of germ-line cells in *Xenopus*, mouse and human [21, 23, 25, 36, 37, Table 3].

A simple analysis of the merged images in which green and red fluorescence are shown simultaneously on one preparation indicates the general activity of a given cell type. The cytoplasm of the germ-line cells appears green so it can be concluded that the inactive mitochondria prevail over the active ones in these cells. In the somatic cells in the merged images much more red color can be seen, which means that the majority of mitochondria in these cells are active. The approach based on dividing the fluorescence spectrum on four classes of intensity allowed us to confirm our conclusions based on the merged images. From this comparison one can see that in the germ-line cells of the gonad inactive mitochondria prevail over the active ones. The active mitochondria that are classified as A4 are rare or absent in these cells. In these precious germ-line cells, for most of the time, A3 is a maximum mitochondrial activity level. They appeared to switch on higher level A4 during periods of enlarged energy requirements like mitotic divisions of oogonia. A4 class of mitochondrial activity was detected also in trophocytes, actively supporting growing oocytes during oogenesis. They degenerate after fulfilling their function and therefore don’t need to protect their DNA from damage. In the somatic cells active mitochondria are on higher activity level than mitochondria in the germ-line cells. Taking into account the above consideration, we can state once more that the stereology used for calculations of the mitochondria of germ-line cells tells us nothing about their metabolic activity.

### Distribution of mitochondria in postvitellogenic oocytes

The distribution of the mitochondria of different fluorescence intensity classes of active and inactive mitochondria in postvitellogenic oocytes of *D*. *veneta* was very interesting. Some of the postvitellogenic oocyte’s mitochondria were dispersed almost regularly within the ooplasm (mainly A1 and N1 classes). The mitochondria N2 were localized more in the direction of the peripheral ooplasm. Whereas organelles emitting JC-1 fluorescence classified in classes A2, A3 and N3 were distributed asymmetrically, creating a crescent like accumulation close to one of the cell poles. Only a few point signals A4 one can observe at the cell’s pole, in the area where there is no connection with somatic cells.

Mitochondria N3 occupied the region close to the A3 crescent but they were much more numerous. It is possible that they would be activated during the oocyte maturation.

Changes in distribution of mitochondria in the germ-line cells depending on the differentiation stage have been found in the oocytes of different mammals, like mouse, human [25, 38, 40, 41], hamster [42], pig [39], cattle [19], and other vertebrates like zebrafish [18] and *Xenopus* [17, 21]. Many authors interpret the mitochondria translocation in the oocytes as their preparation for the egg maturation processes [22, 23, 39, 40, 42]. In many animal species, the processes of egg maturation are connected with the rise of mitochondrial activity [19, 20, 42, 44] because the processes that take place in the egg nucleus and cytoplasm, the modification of cytoskeleton and the production and accumulation of mRNA are necessary preparations for the onset of embryo development and depend on ATP. It is possible that the asymmetric distribution of mitochondria in the vitellogenic oocyte of *D*. *veneta* is connected with the approaching maturation process and accumulation of active mitochondria on one of the cell poles points to the site of future polar body expulsion. A similar accumulation of mitochondria points to the future site of the first polar body extrusion in mouse oocytes [45]. The behavior of mitochondria in ontogeny of animals belonging to different species studied to date is compared in the Table 3.

### Activity of Cytochrome Oxidase and mtDNA replication

Examination of the Cytochrome Oxidase activity in *Xenopus levis* previtellogenic oocytes revealed that the activity of COX diminished in the growing mitochondrial cloud [46, 47]. The authors interpreted this drop in enzyme activity as the inhibition of enzyme synthesis during the replication of mtDNA [47]. Tourte, Mignotte and Mounolou [48] and Mignotte et al. [49] studied the distribution and replicative activity of mitochondria in the same species and found two populations of these organelles. One population stays around the nucleus and actively replicates mtDNA and the second population moves toward the vegetal pole and stops replicating. According to the authors, the second population enters the germ plasm and eventually the primordial germ cells of the progeny. Recently, Kogo et al., [21] while studying mitochondrial activity, the expression of ATP synthase and COX activity in different regions of the oocytes of *X*. *laevis* also found two populations of mitochondria, one of which was dispersed through the entire ooplasm and the second which was distributed within the cortical layer around the vegetal pole. The first was named the “somatic” mitochondria and the second the “germ-line” mitochondria. A similar segregation of mitochondria into two populations was found in oocytes of *Coturnix coturnix* [50, 51]. Moreover, in this case the authors claimed that the mitochondria with inactive COX were destined to enter the primordial germ cells and the mitochondria with a high activity of COX would become included into the somatic cells of the next generation.

The distribution of COX activity in somatic cells and the segregation of mitochondria in the postvitellogenic oocytes of *D*. *veneta* (present paper) confirmed the results obtained using the JC-1 stain. There was strong accumulation of DAB in the mitochondria of somatic cells while in postvitellogenic oocytes only mitochondria that were situated in the peripheral regions of the cells contained DAB deposits whereas the organelles in deeper regions of the ooplasm did not show the enzyme activity.

### Detection of ROS and the antioxidant defense

The destructive influence of oxidative stress has been shown in mouse oocytes [25, 52]. Reactive oxygen species were found in all cell types in the ovaries of *D*. *veneta*. The lowest level of those compounds was found in somatic cells, which correlates with the lowest quantity of mitochondria in these cells. The different content of ROS in particular prooocytes was interesting. The reaction level was higher in the cytoplasm than in the nucleus in some of them, while in the remaining ones it was the opposite. It is possible that these differences represent very early signs in the determination of prooocytes into oocytes or trophocytes. The low level of ROS in the central ooplasm of postvitellogenic oocytes and elevated level of these substances in subplasmalemmal positions is also characteristic. Such a result is in register with JC-1 staining which indicates more active mitochondria in the same cell regions. The reaction showing ROS distribution in the *D*. *veneta* ovary corresponds well with the distribution of mitochondrial SuperOxide Dismutase (MnSOD). Manganese superoxide dismutase is the primary antioxidant enzyme that resides in mitochondria that can protect cells from oxidative damage by catalyzing the dismutation of superoxide to H_2_O_2_ and O_2_. MnSOD is important in maintaining intracellular ROS and redox balance. Increased MnSOD protects tissues against oxidative stress [53, 54]. According to our results, the strongest protection MnSOD is rendered to the oocytes of the species that was studied (they possess the highest expression of the enzyme) and the weakest to the somatic cells.

### The fate of inactive mitochondria in oocytes

The occurrence of two populations of mitochondria in germ-line cells (active and inactive) has been described in many other animal species [17, 19, 21, 23, 55]. What is the biological reason for the existence of inactive mitochondria in the germ-line cells? According to Kogo et al. [21], the separation of an inactive subset of mitochondria in amphibian oocytes is connected with their accurate transmission to the next generation. In other animal groups like mammals, fishes [18], nematodes [20], birds [50, 51] and insects [35], the existence of a fraction of inactive mitochondria in oocytes was also interpreted as being connected with the protection of this mitochondria against mtDNA mutations before they could be transmitted to the progeny [21, 35].

In studying the mitochondria in germ-line cells one can see a great deal of concern about the quality of these organelles when they are transmitted to the next generation [56]. There are two steps in protecting individuals against the transmission of deleterious mutations to the progeny. One is the elimination of damaged mitochondria to the zygote in a mechanism, which is called bottleneck [56, 57, 58, 59, 60, 61, 62, 63, 64, 65]; the second is lowering mitochondrial activity in the germ-line cells in order to minimize the damage to mitochondrial DNA by the reactive oxygen species (ROS) that are produced during oxidative phosphorylation [21, 66]. ROS generate mutations in the mitochondrial DNA (mtDNA) that deteriorates the functions of these organelles [25, 67, 68, 70] and, in addition, is considered to be the main source of aging [37, 71, 72, 73, 74, 75, 76].

The concern about lowering mitochondrial activity in germ-line cells is so high that a part of ATP is produced in oocytes anaerobically [23, 36, 55] or transferred to the oocytes from follicular cells [19, 34, 37, 77].

It is well established that there is a strong positive correlation between membrane potential and ROS production. At high membrane potentials, even a small increase in the membrane potential gives rise to a large stimulation of H_2_O_2_ production [78]. Similarly, only a small decrease in membrane potential is capable of inhibiting H_2_O_2_ production [78, 79]. Therefore, ‘mild uncoupling’, i.e. a small decrease in membrane potential, was suggested to have a natural antioxidant effect [68]. Thus, lowering OXPHOS in the germ-line cells mitochondria lowers or even stops ROS production and protects their mtDNA against mutational degradation. The present paper, which describes the prevalence of inactive mitochondria in the germ-line cells of *D*. *veneta*, corroborates the general tendency to protect these organelles from destruction and to keep them fit for future generations that has been found in the majority of species studied to date. Moreover, a higher expression of MnSOD in the mitochondria of germ-line cells additionally protects them from an accumulation of mutated mtDNA.
